# Pubertal Testicular Function in Childhood Cancer Survivors and Future Fertility

**DOI:** 10.1001/jamanetworkopen.2026.38294

**Published:** 2026-10-09

**Authors:** Mikael Koskela, Kathleen Duffin, Melanie Korhonen, Mikko Anttonen, Hanne Frederiksen, Anders Juul, Rod T. Mitchell, Kirsi Jahnukainen

**Affiliations:** 1 Children’s Hospital, University of Helsinki and Helsinki University Hospital, Helsinki, Finland; 2 Department of Women’s and Children’s Health, Karolinska Institute and University Hospital, Stockholm, Sweden; 3 Centre for Reproductive Health, Institute of Regeneration and Repair, University of Edinburgh, Edinburgh, United Kingdom; 4 Royal Hospital for Children and Young People, Edinburgh, United Kingdom; 5 Department of Clinical Chemistry, University of Helsinki and Helsinki University Hospital, Helsinki, Finland; 6 Department of Growth and Reproduction, Copenhagen University Hospital–Rigshospitalet, Copenhagen, Denmark; 7 International Research Centre for Endocrine Disruption of Male Reproduction and Child Health (EDMaRC), Copenhagen University Hospital–Rigshospitalet, Copenhagen, Denmark; 8 Department of Clinical Medicine, University of Copenhagen, Copenhagen, Denmark

## Abstract

**Importance:**

Childhood cancer treatment can result in male infertility. Understanding which patients are at risk of future infertility would facilitate early fertility counseling.

**Objectives:**

To investigate associations between pubertal reproductive hormone levels and adult gonadal outcomes and cancer treatment exposures.

**Design, Setting, and Participants:**

This cohort study was conducted at Helsinki Children’s Hospital among male childhood cancer survivors who received a diagnosis under the age of 17 years between 1964 and 2000, for whom at least 1 reproductive hormone concentration (measured 1982-2017) was available. Data were analyzed from May to December 2025.

**Exposure:**

Alkylating chemotherapy, quantified using cyclophosphamide equivalent dose (CED), and testicular radiotherapy.

**Main Outcomes and Measures:**

Serum hormone levels (follicle-stimulating hormone [FSH], luteinizing hormone [LH], or testosterone) were collected at 2-year intervals between ages 12 and 18 years and at age 20 or older; hormone concentrations were converted to age-specific *z* scores. Data on gonadal outcomes were obtained in adulthood.

**Results:**

The study included 221 childhood cancer survivors (median age at diagnosis, 6.0 years [IQR, 3.2-11.7 years]). FSH showed a dose-dependent pattern with testicular radiotherapy, with elevated FSH *z* scores seen from age 12 years among patients who received more than 1 Gy of testicular radiotherapy. Compared with the group that received less than 4 g/m^2^ CED (median FSH *z* score, 0.1; IQR, −1.2 to 1.2) at age 18 years, higher FSH *z* scores occurred among patients who received 4 to less than 12 g/m^2^ CED (median FSH *z* score, 1.4; IQR, 0.5-2.6; *P* = .04) or received 12 g/m^2^ or more CED (median FSH *z* score, 1.6; IQR, 0.7-2.5; *P* = .007). There was evidence of normalization at age 20 years or older for patients who received 4 to less than 12 g/m^2^ CED (median FSH *z* score, 0.7; IQR, −0.6 to 1.7) but not those who received 12 g/m^2^ or more CED (median FSH *z* score, 1.7; IQR, 0.9-2.5). The highest FSH *z* score at age 14 to 16 years was associated with azoospermia in adulthood, with an optimal cutoff corresponding to 11.2 IU/L at age 14 years and 12.5 IU/L at age 16 years (area under the curve, 0.87). Elevated LH *z* scores occurred from age 12 years onward among patients receiving more than 14 Gy of radiotherapy, but exposure to high CED was not associated with impaired Leydig cell function.

**Conclusions and Relevance:**

In this cohort study of male patients treated for childhood cancer, gonadotrophin levels during puberty were associated with future testicular function. Impairment of testicular function after radiotherapy persisted into adulthood, while there was evidence of recovery after alkylating chemotherapy. The results suggest increased sensitivity of the Sertoli cell–germ cell compartment compared with Leydig cells. These findings can be used to guide reproductive counseling for male survivors of childhood cancer.

## Key Points

**Question:**

Among male childhood cancer survivors, are reproductive hormone levels during puberty associated with fertility potential and testosterone production in adulthood?

**Findings:**

In this cohort study involving 221 male childhood cancer survivors, follicle-stimulating hormone (FSH) measurements during puberty among patients receiving testicular radiotherapy were associated with future azoospermia, with an optimal cutoff corresponding to 11.2 IU/L at age 14 years and 12.5 IU/L at age 16 years. For those receiving intermediate-dose alkylating chemotherapy, increased FSH in puberty often resolved and Leydig cell function was maintained in adulthood.

**Meaning:**

The findings suggest that gonadotrophin levels during puberty are associated with future testicular function among childhood cancer survivors.

## Teaser

This cohort study assesses the association between childhood cancer treatment exposure and reproductive hormone values measured from puberty to adulthood and the association between hormone levels and adult gonadal outcomes among male survivors of childhood cancer.

## Introduction

Male fertility depends on spermatogenesis, the process where germ cells mature from immature spermatogonia through to spermatozoa, which is supported by the Leydig and Sertoli cells of the testis and regulated by the hypothalamic-pituitary-gonadal axis.^1^ Childhood cancer treatment may cause damage to any part of this pathway,^1^ reducing later fertility capacity; this is particularly important given that over 80% of children in Europe with a diagnosis of cancer will go on to become long-term survivors.^2,3^ Leydig cells, responsible for production of testosterone, are believed to be more resistant than germ cells to therapy-induced damage.^4,5^ With functioning Leydig cells, patients are likely to produce normal levels of testosterone and progress through puberty even in the absence of spermatogenesis. As such, pubertal progression is not necessarily indicative of unimpaired fertility.

Future fertility is a source of concern for patients with cancer in childhood and their families,^6,7^ and eligible patients should be offered fertility preservation prior to undergoing gonadotoxic treatment.^8^ Specific treatments, including alkylating chemotherapy and gonadal radiotherapy, are considered to pose a significant risk to fertility.^9^ Given these patients are undergoing treatment in childhood, it may take years or even decades for any impact of treatment on fertility to manifest. It is therefore necessary to consider methods of assessment during puberty that might correlate with future gonadal function. Follicle-stimulating hormone (FSH) levels in adulthood are associated with sperm production, thereby serving as an indirect measurement of fertility.^10^ However, little is known on the association between pubertal reproductive hormone levels after exposure to cancer therapies and gonadal outcomes in adulthood. There are also limited data correlating alkylating agent exposure with longer-term reproductive outcomes, which is reflected in the large variability between centers in defining eligibility for testicular tissue cryopreservation, the currently experimental fertility preservation technique for prepubertal males.^11^

Understanding which patients are at risk of future infertility would facilitate early provision of appropriate fertility counseling. In this study, we examined the association between childhood cancer treatment exposure and reproductive hormone values measured from puberty to adulthood, using normative reference datasets for comparison. We also analyzed the association between hormone levels and adult gonadal outcomes, including azoospermia and oligospermia, testicular volume, paternity, and use of testosterone replacement therapy (TRT).

## Methods

### Study Design

This study is part of a research project on health and fertility outcomes among male childhood cancer survivors who were treated at younger than 17 years at the Helsinki Children’s Hospital between 1964 and 2000 (survival, ≥5 years).^12,13,14,15^ The cohort included patients with all childhood cancer diagnoses, except those with brain tumors, because they underwent cancer treatment in a different hospital. The present analysis included 221 survivors with at least 1 available hormone (FSH, luteinizing hormone [LH], or testosterone) measurement during pediatric follow-up or in adulthood (eFigure 1 in Supplement 1). This study followed the Strengthening the Reporting of Observational Studies in Epidemiology (STROBE) reporting guideline and was approved by the research ethics committee of Helsinki University Hospital and The Finnish Social and Health Data Permit Authority. The study used only anonymized registry-based data; according to national policy, informed consent was therefore not required.

### Data Collection

Hospital records provided data on serum hormone measurements (FSH, LH, and testosterone), semen samples in adulthood, testicular volumes, need for TRT during puberty, and cancer treatment details. Semen samples with more than 0 to less than 15 million/mL sperm concentration were classified as oligospermic. In addition, using Finnish personal identification numbers, survivors were linked with national registry data. The Digital and Population Data Services Agency (accessed December 31, 2014) provided data on survivors’ offspring. The National Prescription Register (accessed December 31, 2020) provided data on testosterone purchases in adulthood (Anatomical Therapeutic Classification System Code G03BA03). After retrieval from the registries, data were pseudoanonymized; the research group had access only to unidentifiable registry data. Cumulative cyclophosphamide equivalent dose (CED) served as a marker of alkylating agent exposure.^16^ The Late Effects Group at the University of Texas MD Anderson Cancer Center calculated organ-specific radiotherapy doses for the testes and pituitary gland, as previously described.^17^ Serum hormone levels and testicular volume measurements were collected after the end of cancer therapy at 2-year intervals between ages 12 and 18 years and in adulthood (age ≥20 years). If multiple hormone samples or testicular volume measurements were available in adulthood, the latest was included in the analyses. We excluded all serum hormone measurements, semen samples, and testicular volume measurements obtained during ongoing TRT.

### Laboratory Methods and Reference Materials

Serum concentrations of FSH, LH, and testosterone were measured using routine clinical laboratory methods available at the time of sampling (measured in 1982-2017). Testosterone measurements were performed using liquid chromatography–tandem mass spectrometry from 2005 onward. Prior to 2005, testosterone was analyzed using an in-house chromatographic method combined with radioimmunoassay, which has been validated to yield results comparable with that of liquid chromatography–tandem mass spectrometry.^18^ FSH and LH were measured using the Wallac AutoDELFIA system (PerkinElmer) until June 2011. From June 2011 to May 2016, assays were carried out on the Roche Modular platform, which yielded systematically 15% to 30% higher values compared with the Wallac AutoDELFIA system. Between May 2016 and November 2019, the Abbott Architect system was used, providing results slightly lower (<10%) than those obtained with the Roche Modular platform.

Hormone concentrations were converted into *z* scores using 2 normative reference datasets. Reference material 1 is based on measurements from healthy Danish individuals (samples collected between 1991 to 2018), and it provides age-specific *z* scores for FSH, LH, and testosterone from birth to 80 years.^19,20^ Owing to the lack of age-specific FSH references between 20 and 40 years, FSH *z* scores at age 20 years or older (median, 25 years [IQR, 20-28 years]) were calculated using the 20-year reference values. Reference material 2, based on measurements from healthy Norwegian children and adolescents (samples collected between 2010 and 2016), provides age-specific *z* scores for FSH and LH between 6 and 16 years and for testosterone between 6 and 18 years.^21^ Testicular length and width were measured with a ruler, and testicular volume was calculated using the following formula: π/6 × length × width^2^. The volumes were then converted into *z* scores using age-specific references, as previously described.^13,22^ Data on gonadal outcomes (testicular volume, semen quality, and paternity) and adult hormone levels have been included in previous reports,^12,13^ whereas the present study is the first to report pubertal hormone values and normalized adult data.

### Statistical Analysis

Statistical analysis was conducted from May to December 2025. Normalized hormone levels at age 12 to 18 years and age 20 years or older were analyzed in association with cancer treatment exposures (testicular radiotherapy dose and CED) and 4 adulthood outcomes: semen analysis (sample age ≥20 years), testosterone prescriptions (age ≥20 years), paternity, and low testicular volume (*z* score below −2 at age ≥20 years). Group differences in hormone levels were assessed using the Mann-Whitney *U* test (2 groups) or Kruskal-Wallis test (>2 groups). If overall difference was significant in the Kruskal-Wallis test, post hoc pairwise comparisons were performed with the Mann-Whitney *U* test applying Holm correction for multiple testing. Associations between TRT (initiated during pediatric surveillance or in adulthood) and treatment exposures were evaluated by logistic regression, yielding odds ratios (ORs) with 95% CIs. Correlations between continuous variables were examined using Pearson correlation coefficient. By calculating areas under the curves (AUCs) and Youden indices (optimal cutoff point), receiver operating characteristic (ROC) analyses served as a method to assess the discriminative ability of hormone *z* scores for azoospermia, adult TRT (age ≥20 years), nonpaternity, and low adult testicular volume. Statistical analyses were conducted with R, version 4.1.2 (R Project for Statistical Computing). All *P* values were from 2-sided tests and results were deemed statistically significant at *P* < .05.

## Results

### Study Cohort Characteristics and Comparison Between Reference Materials

The study included 221 childhood cancer survivors; median age at diagnosis was 6.0 years (IQR, 3.2-11.7 years), and 151 (70.6%) had hematologic malignant neoplasms (Table). Serum hormone measurements were available from 71 to 116 childhood cancer survivors at each time point (eFigure 1 in Supplement 1), with a median of 2 samples (IQR, 1-3) per patient. Pubertal hormone *z* scores were derived using reference materials 1 and 2. FSH *z* scores differed slightly between the reference materials, while LH and testosterone *z* scores were nearly identical (eFigures 2-4 in Supplement 1). Absolute hormone concentrations by treatment exposures are shown in eTable 1 in Supplement 1.

**Table.** Patient and Treatment Characteristics and Gonadal Outcomes in Male Childhood Cancer Survivors With Available Hormone Samples

Characteristic	Patients (N = 221)
Age at diagnosis, median (IQR), y	6.0 (3.2-11.7)
Diagnoses, No. (%)	
Hematologic malignant neoplasms	156 (70.6)
Sarcoma, germinoma, neuroblastoma	43 (19.5)
Other tumors^a^	22 (10.0)
Diagnosis period, No. (%)	
1964-1979	61 (27.6)
1980-1989	105 (47.5)
1990-2000	55 (24.9)
Cumulative testicular radiotherapy dose, No. (%), Gy^b^	
0	96 (43.4)
>0 to <1	62 (28.1)
1 to 14	40 (18.1)
>14	21 (9.5)
Data unavailable	2 (0.9)
CED, No. (%), g/m^2^	
0	62 (28.1)
>0 to <4	42 (19.0)
≥4 to <12	50 (22.6)
≥12	65 (29.4)
Data unavailable	2 (0.9)
Cumulative pituitary radiotherapy dose, No. (%), Gy^c^	
<20	164 (74.2)
≥20	55 (24.9)
Data unavailable	2 (0.9)
HSCT, No. (%)	
No	176 (79.6)
Yes	43 (19.5)
Data unavailable	2 (0.9)
Time from end of therapy to hormone measurement, median (IQR), y	
Age 12 y	4.6 (1.8-6.2)
Age 14 y	6.3 (2.9-8.8)
Age 16 y	6.3 (2.0-10.2)
Age 18 y	5.4 (2.6-11.5)
Age ≥20 y^d^	14.6 (9.6-18.8)
Semen samples in adulthood (≥20 y) (n = 87)	
Normospermia, No. (%)	43 (49.4)
Oligospermia, No. (%)	16 (18.4)
Azoospermia, No. (%)	28 (32.2)
Age at sample, median (IQR), y	25.2 (22.3-30.4)
TRT in adulthood (≥20 y)^e^ (n = 219)	
TRT, No. (%)	40 (18.3)
Age at evaluation, mean (SD), y	43.2 (7.6)
Paternity (n = 219)	
Registered children, No. (%)	87 (39.7)
Age at evaluation, mean (SD), y	37.6 (7.4)
Testicular volume measurements in adulthood (≥20 y) (n = 42)	
Low adult testicular volume, No. (%)^f^	12 (28.6)
Age at evaluation, mean (SD), y	27.7 (4.1)

Abbreviations: CED, cyclophosphamide equivalent dose; HSCT, hematopoietic stem cell transplantation; TRT, testosterone replacement therapy.

^a^ Does not include brain tumors.

^b^ Median testicular radiotherapy dose in the group receiving less than 1 Gy was 0.1 Gy (IQR, 0.04-0.2 Gy); in the group receiving 1 to 14 Gy, 10 Gy (IQR, 10-11 Gy); and in the group receiving more than 14 Gy, 24 Gy (IQR, 24-24 Gy).

^c^ Median pituitary radiotherapy dose in the group receiving less than 20 Gy was 0.1 Gy (IQR, 0-10 Gy); and in the group receiving 20 Gy or more, 24 Gy (IQR, 24-24 Gy).

^d^ Median (IQR) age for this group was 25 (20-28) years.

^e^ Denotes having used testosterone at age 20 years or older.

^f^ Denotes *z* score below −2.

### Association Between Cancer Treatment Exposures and Hormone Levels

FSH *z* scores were elevated above the normal reference range from age 12 years onward among patients exposed to testicular radiotherapy doses greater than 14 Gy (median dose, 24 Gy; IQR, 24-24 Gy); increased values were also consistently seen among those receiving doses of 1 to 14 Gy (median dose, 10 Gy; IQR, 10-11 Gy) (Figure 1A). In patients who did not undergo radiotherapy or those exposed to only low levels of scattered testicular radiotherapy (<1 Gy), FSH levels were within reference range throughout follow-up among patients who received less than 4 g/m^2^ CED. Compared with the group that received less than 4 g/m^2^ CED (median FSH *z* score, 0.1; IQR, −1.2 to 1.2), at age 18 years FSH *z* scores were increased among those who received 4 to less than 12 g/m^2^ CED (median FSH *z* score, 1.4; IQR, 0.5-2.6; *P* = .04) or received 12 g/m^2^ or more CED (median FSH *z* score, 1.6; IQR, 0.7-2.5; *P* = .007); there was evidence of normalization of FSH *z* scores at age 20 years or older for patients who received 4 to less than 12 g/m^2^ CED (median FSH *z* score, 0.7; IQR, −0.6 to 1.7) but not those who received 12 g/m^2^ or more CED (median FSH *z* score, 1.7; IQR, 0.9-2.5) (Figure 1B).

**Figure 1.**
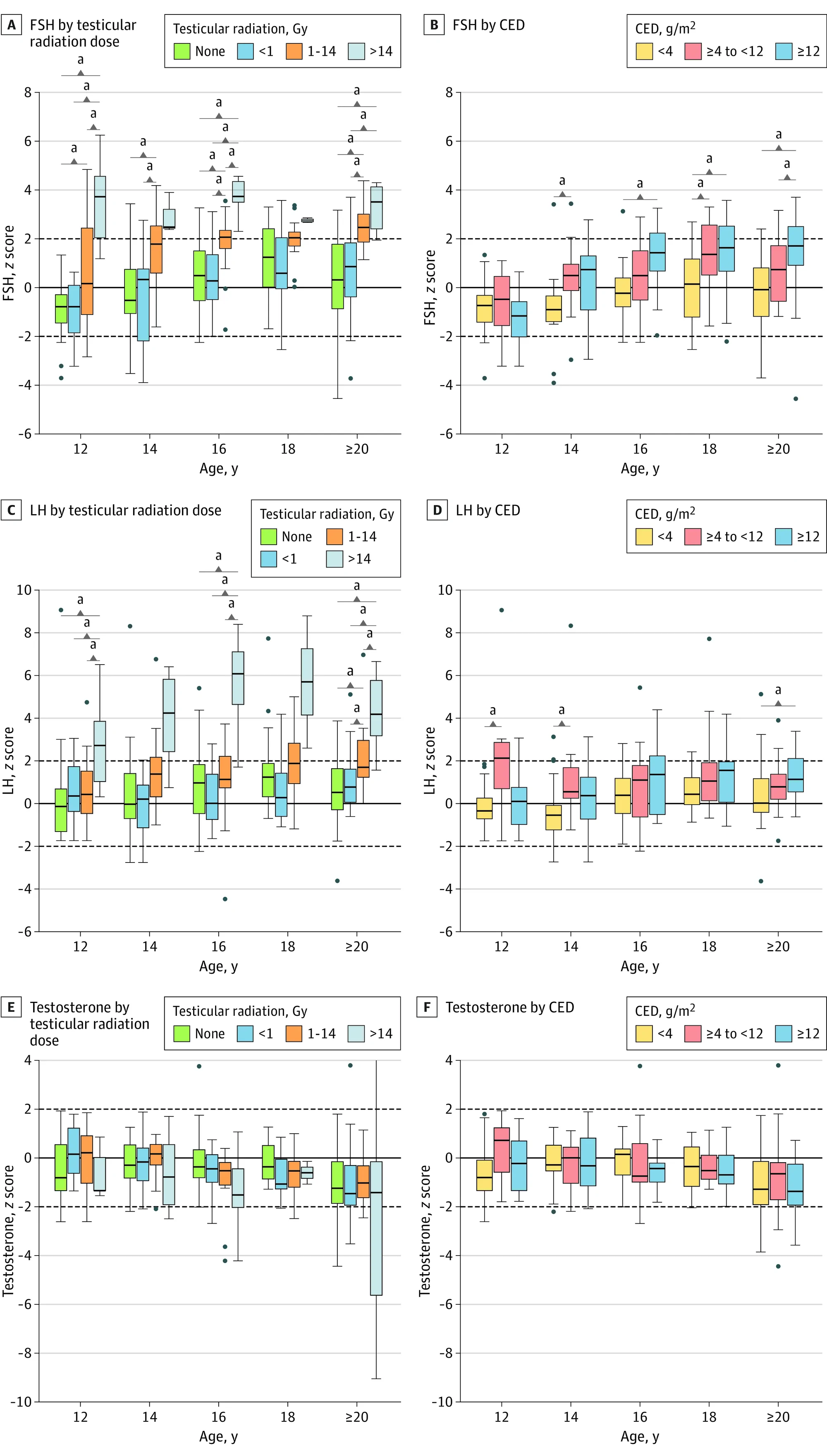
Box Plots of Hormone Values Among Male Childhood Cancer Survivors Stratified by Testicular Radiation Dose and Cyclophosphamide Equivalent Dose (CED) Patients who received testicular radiotherapy doses of 1 Gy or more were excluded from the analyses involving CED. Hormone values were standardized to age-specific *z* scores using reference material 1. The area between the 2 horizontal dashed lines indicates normal reference range. The median age at measurement in the group aged 20 years or older was 25 years (IQR, 20-28 years). Number of patients with available hormone sample in each group is presented in eTable 3 in Supplement 1. Horizontal lines (inside the boxes) denote the median value. The dots indicate outlier values occurring beyond 1.5 times the IQR, with measurement (of the 1.5 times the IQR) starting from the lower or upper quartile. FSH indicates follicle-stimulating hormone; LH, luteinizing hormone. ^a^*P* < .05. Significant differences between groups are presented in eTable 1 in Supplement 1.

LH *z* scores were increased from age 12 years onward among patients receiving more than 14 Gy of testicular radiotherapy. A significant difference between those receiving 1 to 14 Gy (median *z* score, 1.7; IQR, 1.3-3.0) and those receiving less than 1 Gy (median *z* score, 0.8; IQR, 0.1-1.6) was observed only at age 20 years or older (*P* = .02) (Figure 1C). When patients who received 1 Gy or more testicular radiotherapy were excluded, LH levels remained predominantly within the reference range at all time points among all CED groups (Figure 1D). Testosterone *z* scores showed no marked variation across testicular radiotherapy or CED groups (Figure 1E and 1F). Results for FSH, LH, and testosterone remained largely unchanged after exclusion of patients who received 20 Gy or more of pituitary radiotherapy (eFigure 5 in Supplement 1).

### Risk Factors for TRT

In the full survivor cohort, which included additionally those without hormone measurements (n = 255; eFigure 1 in Supplement 1), 80 childhood cancer survivors (31.4%) received TRT during puberty or adulthood. The use of TRT was associated with testicular radiotherapy at doses of 1 to 14 Gy (OR, 3.13; 95% CI 1.44-6.92) and more than 14 Gy (OR, 126.28; 95% CI, 22.68-2401.08), whereas 4 to less than 12 g/m^2^ CED (OR, 1.76; 95% CI, 0.78-3.98), 12 g/m^2^ or more CED (OR, 1.77; 95% CI, 0.82-3.90), and pituitary radiotherapy (OR, 1.03; 95% CI, 0.43-2.33) showed no significant association with TRT (eTable 2 in Supplement 1).

### Correlation Between Hormone Levels and Testicular Volumes

eFigure 6 in Supplement 1 presents correlations between hormone levels and testicular volumes when measured at the same time point. FSH and LH *z* scores showed significant negative correlation at age 16 years (FSH: *r* = –0.48; *P* < .001; LH: *r* = –0.38; *P* = .002), 18 years (FSH: *r* = –0.56; *P* < .001; LH: *r* = –0.39; *P* = .003), and 20 years or older (FSH: *r* = –0.61; *P* < .001; LH: *r* = –0.59; *P* < .001).

### Association Between Hormone Levels and Adulthood Gonadal Outcomes

Figures 2 and 3 show associations between hormone levels at different time points and gonadal function in adulthood. FSH *z* scores were consistently increased among patients with azoospermia, whereas they remained mainly within the normal reference range among patients with oligospermia; compared with the normospermia group (median FSH *z* score, −0.4; IQR, −1.1 to 0.6), patients with oligospermia (median FSH *z* score, 1.2; IQR, 0.5-1.8; *P* = .001) had significantly higher FSH *z* scores only at age 20 years or older. LH *z* scores were elevated above the reference range from age 14 years onward among those using TRT. Testosterone *z* scores showed little variation across the gonadal outcome groups.

**Figure 2.**
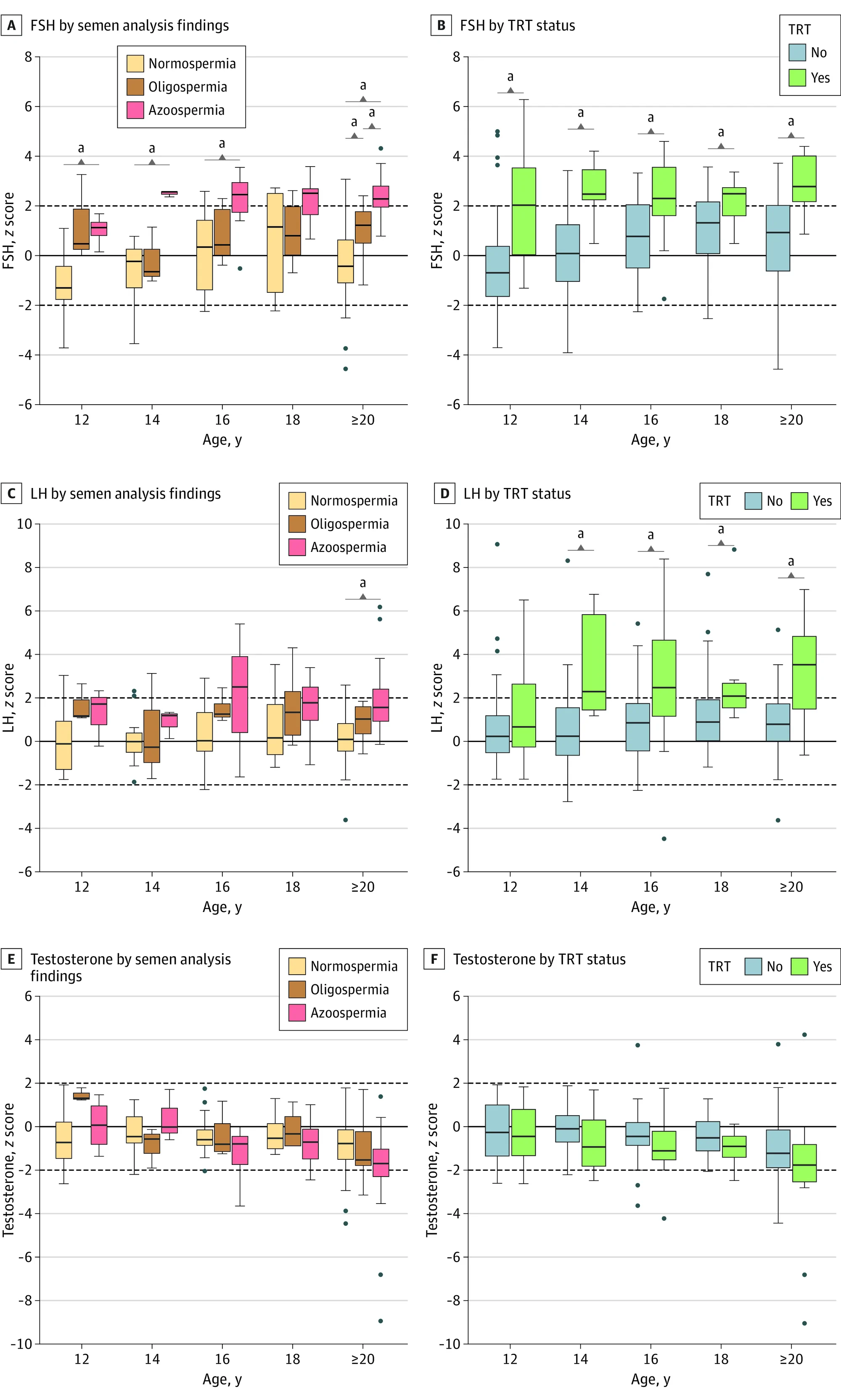
Box Plots of Hormone Values Among Male Childhood Cancer Survivors Stratified by Semen Analysis Findings and Testosterone Replacement Therapy (TRT) Status Hormone values were standardized to age-specific *z* scores using reference material 1. The area between the 2 horizontal dashed lines indicates normal reference range. The median age at measurement in the group aged 20 years or older was 25 years (IQR, 20-28 years). Number of patients with available hormone sample in each group is presented in eTable 3 in Supplement 1. Horizontal lines (inside the boxes) denote the median value. The dots indicate outlier values occurring beyond 1.5 times the IQR, with measurement (of the 1.5 times the IQR) starting from the lower or upper quartile. FSH indicates follicle-stimulating hormone; LH, luteinizing hormone. ^a^*P* < .05.

**Figure 3.**
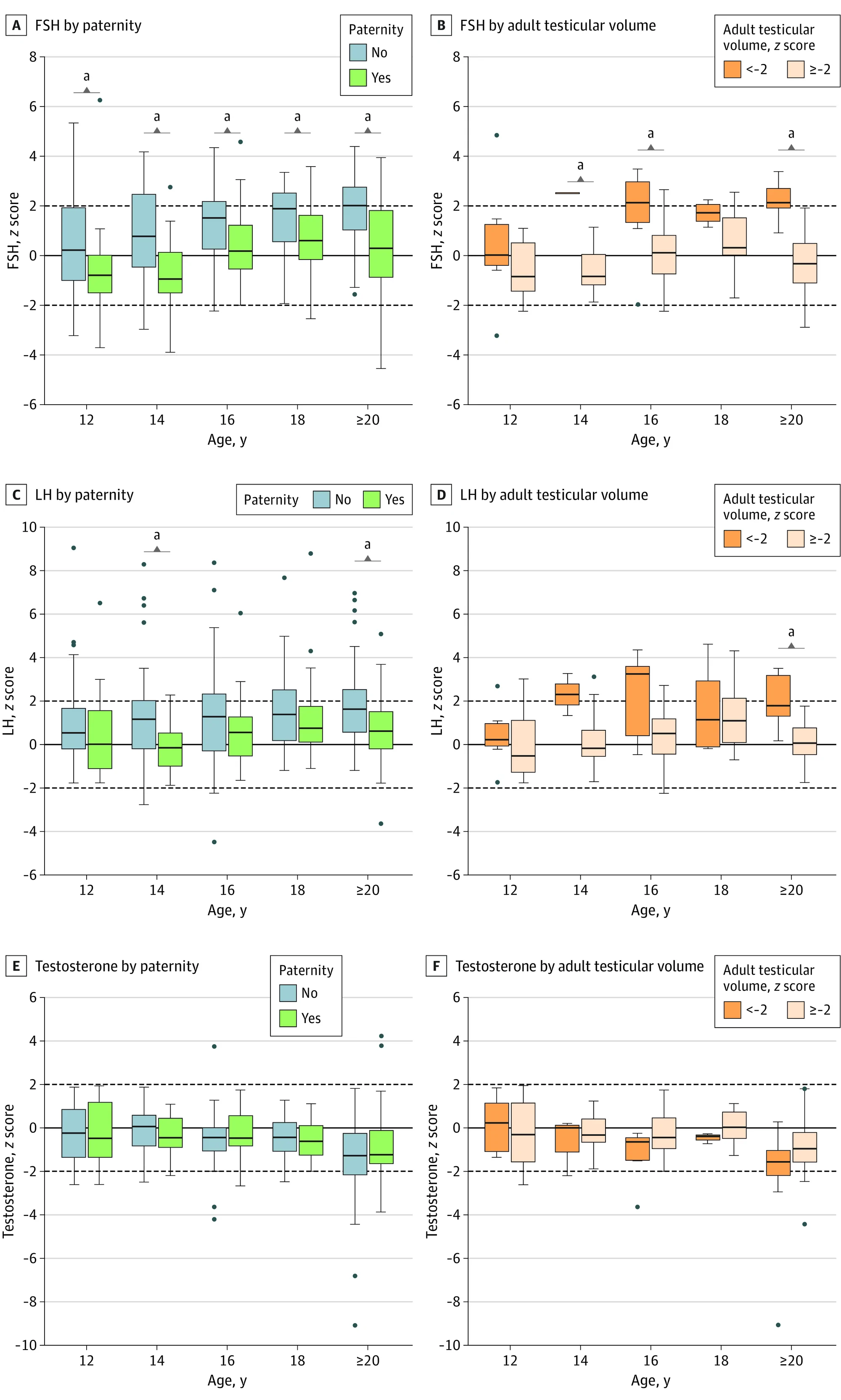
Box Plots of Hormone Values Among Male Childhood Cancer Survivors Stratified by Paternity and Adult Testicular Volume Hormone values were standardized to age-specific *z* scores using reference material 1. The area between the 2 horizontal dashed lines indicates normal reference range. The median age at measurement in the group aged 20 years or older was 25 years (IQR, 20-28 years). Number of patients with available hormone sample in each group is presented in eTable 3 in Supplement 1. Horizontal lines (inside the boxes) denote the median value. The dots indicate outlier values occurring beyond 1.5 times the IQR, with measurement (of the 1.5 times the IQR) starting from the lower or upper quartile. FSH indicates follicle-stimulating hormone; LH, luteinizing hormone. ^a^*P* < .05.

Figure 4 provides ROC analyses for FSH and LH values (highest *z* score at age 14-16 years) in identifying patients with azoospermia and those using TRT, respectively. FSH detected azoospermia with an AUC of 0.87; the optimal cutoff was at a *z* score of 1.8, corresponding to 11.2 IU/L at age 14 years and 12.5 IU/L at age 16 years. LH identified patients using TRT with an AUC of 0.75. In addition, eFigure 7 in Supplement 1 provides ROC analyses for FSH and LH values (highest *z* score at age 14-16 years) in identifying patients with nonpaternity and low adult testicular volume. Associations between FSH and azoospermia and between LH and TRT using hormone values at each time point alone are provided in eFigure 8 and eFigure 9 in Supplement 1.

**Figure 4.**
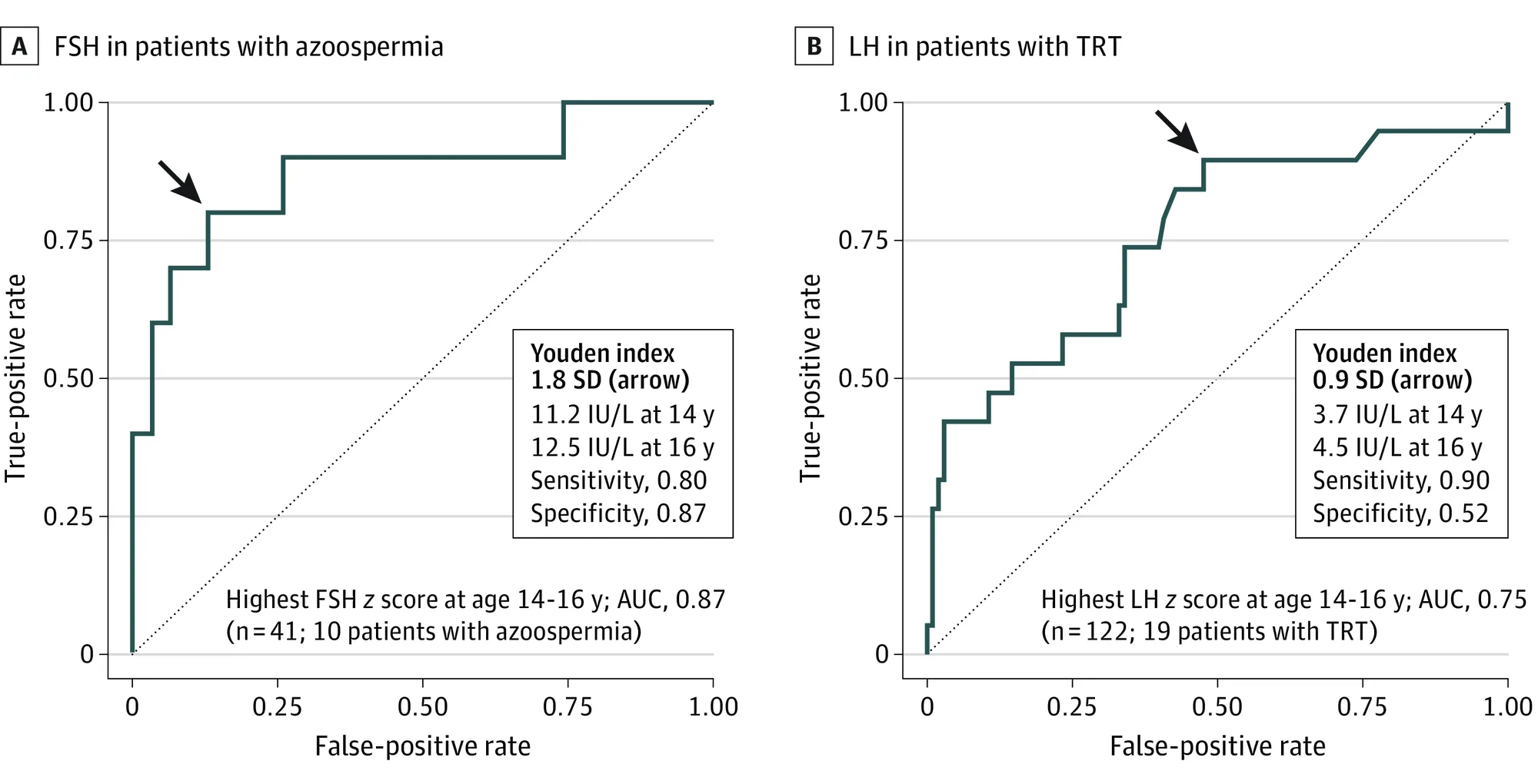
Receiver Operating Characteristic Curves and Corresponding Youden Indices With Optimal Cutoff Points for Identifying Patients 20 Years or Older With Azoospermia and With Testosterone Replacement Therapy (TRT) Hormone values were standardized to age-specific *z* scores using reference material 1. AUC indicates area under the curve; FSH, follicle-stimulating hormone; LH, luteinizing hormone.

## Discussion

Exposure to radiotherapy and chemotherapy during childhood are recognized causes of impaired fertility among males.^8^ However, to our knowledge, the value of monitoring testicular function during puberty to identify potential for recovery after childhood cancer treatment has not been described. The present study provides longitudinal data on testicular function during puberty in a cohort of children receiving cancer treatment. We show that impaired testicular function after chemotherapy and radiotherapy can be recognized during puberty; and while persistent impairment of testicular function is frequently seen after radiotherapy, recovery in adulthood may occur after alkylating chemotherapy.

FSH levels in adulthood correlate with azoospermia,^10^ but the association has been unclear when FSH is measured during puberty. The present study shows that elevated FSH during puberty was associated with azoospermia in adulthood. Patients with azoospermia had consistently increased FSH at all time points during puberty, and an FSH of 11.2 IU/L at age 14 years and 12.5 IU/L at age 16 years were the optimal cutoff points in identifying patients with azoospermia. These values are slightly higher than the FSH threshold (10.4 IU/L) previously reported in a meta-analysis of childhood cancer survivors, when FSH levels were measured in adulthood.^10^

Testicular volume was poorly correlated with serum FSH during puberty, suggesting that measuring testicular volume in adolescence is less useful at estimating azoospermia in adulthood. In contrast, testicular volume in adulthood correlated well with serum FSH and adult gonadal function.^13^ These findings are consistent with a previous study showing that a substantial proportion of childhood cancer survivors with reduced testicular volume during puberty subsequently achieved normal adult testicular volume.^13^ Our findings are also consistent with previous results on high FSH levels after age 12 years being associated with azoospermia in adulthood after hematopoietic stem cell transplantation in childhood.^23^ Although FSH was associated with azoospermia, it showed poor correlation with paternity. This is likely to be the result of the multifactorial nature of paternity, which is also influenced by factors other than spermatogenesis.

Exposure to scatter-dose radiotherapy (<1 Gy) was not associated with an increased FSH at any time point, while the moderate increase in FSH after 1 to 14 Gy could already be seen at 12 years of age. Exposure to more than 14 Gy was associated with a large increase in FSH, which again was already present during puberty. Therefore, among individuals receiving radiotherapy, the results demonstrated the potential benefit associated with commencing measurement of serum gonadotrophins starting at age 12 years in identifying patients with azoospermia in adulthood.

Exposure to alkylating chemotherapy during childhood has been associated with impaired fertility in adulthood.^24^ In the present study, less than 4 g/m^2^ CED was not significantly associated with spermatogenesis based on FSH measurements, while doses of 4 g/m^2^ or more were associated with a compensatory increase in FSH levels during late puberty, which appeared to normalize by adulthood when the cumulative CED was below 12 g/m^2^. It was only at the very highest CED (≥12 g/m^2^) that increased FSH persisted into adulthood. These data provide further support for the recent European Society of Human Reproduction and Embryology Best Practice recommendation, which states that testicular biopsy for fertility preservation is not recommended for patients who receive less than 4 g/m^2^ CED, and testicular tissue cryopreservation should be offered to those receiving 12 g/m^2^ or more CED.^25^ For individuals receiving 4 to less than 12 g/m^2^ CED, current recommendations state that testicular tissue cryopreservation should be considered.^25^ For this group, our data suggest that despite FSH levels being elevated during puberty, normalization may occur by early adulthood. The possibility for recovery also suggests that caution should be applied when counseling patients receiving 4 to less than 12 g/m^2^ CED about future fertility based on elevated FSH during puberty.

The present data provide further confirmation that Leydig cell function and testosterone production are, compared with the seminiferous epithelium and hence fertility, relatively protected by damage induced by cancer therapy.^4,5^ Elevated LH was seen throughout puberty in the patient cohort exposed to more than 14 Gy of testicular radiotherapy, while a significantly increased LH in the cohort receiving 1 to 14 Gy was seen only in adulthood. Despite this finding, testosterone levels remained largely in the normal range. Therefore, for most patients with increased LH, this would represent a compensated Leydig cell failure. These results are also reflected in the use of exogeneous testosterone, for which the OR was moderately increased among those exposed to 1 to 14 Gy of radiotherapy and largely increased after exposure to more than 14 Gy. In the absence of significant (≥1 Gy) radiotherapy exposure, most patients exposed to alkylating agents (including ≥12g/m^2^ CED) demonstrated normal Leydig cell function during puberty, with LH and testosterone within reference ranges into adulthood. CED was also not associated with use of TRT, although our study may have been underpowered to detect a subtly increased risk.

### Strengths and Limitations

This study has some strengths. A key strength is the use of 2 independent reference materials that provide robust normative data for standardizing the results. The reference materials demonstrated similar values for LH and testosterone, while slight differences occurred in the FSH references. Taken together, these results increased the confidence in the accuracy of the data and potential for applicability across different populations. The use of longitudinal data ensured that markers of testicular function during puberty could be linked with adult reproductive outcomes in the same individual.

This study also has some limitations. Because of the methodological changes in measuring FSH and LH levels during the study period, absolute hormone concentrations should be interpreted with caution. Despite this study being the largest longitudinal follow-up of testicular function during puberty among childhood cancer survivors, some of the groups had a relatively low number of patients. Limited sample size also did not allow for stratification into narrower ranges of radiotherapy doses. The study concentrated on testicular radiotherapy and CED, the most likely gonadotoxic therapies, but it is possible that other treatment-related factors may have influenced the outcome. Temporal changes in semen quality among Finnish men^26^ may have influenced our findings on oligospermia, as our data included semen samples collected over several decades. Hormone levels may have gradually changed over time, which may have influenced the calculated *z* scores. Correlation and ROC analyses were intended to generate hypotheses and should be regarded as exploratory.

## Conclusions

In this cohort study of male childhood cancer survivors, testicular function during puberty was associated with fertility potential and requirement for testosterone in adulthood. In particular, testicular radiotherapy was associated with increased FSH during puberty and subsequent high risk of azoospermia in adulthood. For those exposed to a high CED, the increased FSH during puberty may also persist into adulthood. However, for those exposed to intermediate CED, increased FSH may normalize in adulthood, indicating potential for recovery. In contrast, exposure to a high CED was not associated with impaired Leydig cell function. These results demonstrate the increased sensitivity of the testis to radiotherapy compared with chemotherapy, and the increased sensitivity of the Sertoli cell–germ cell compartment compared with Leydig cells. The findings provide a framework for monitoring testicular function during puberty as a means to assess fertility and testosterone production in adulthood among survivors of childhood cancer.

## Notes

Supplement 1. **eTable 1.** Absolute Hormone Concentrations in Male Childhood Cancer Survivors Treated at the Helsinki Children’s Hospital Between 1964–2000, Stratified by Cancer Treatment Exposures **eTable 2.** Multivariable Logistic Regression Analyses of Risk Factors for Testosterone Replacement Therapy in Male Childhood Cancer Survivors **eTable 3.** Number of Patients at Each Timepoint in Analyses Illustrated in Figures 1–3 **eFigure 1.** Flow Chart of the Patient Selection Process **eFigure 2.** Follicle-Stimulating Hormone (FSH) Z-Scores at Age 12–16 Years, Stratified by (A) Testicular Radiation Dose and (B) Cyclophosphamide Equivalent Dose (CED) **eFigure 3.** Luteinizing Hormone (LH) Z-Scores at Age 12–16 Years, Stratified by (A) Testicular Radiation Dose and (B) Cyclophosphamide Equivalent Dose (CED) **eFigure 4.** Testosterone Z-Scores at Age 12–18 Years, Stratified by (A) Testicular Radiation Dose and (B) Cyclophosphamide Equivalent Dose (CED) **eFigure 5.** (A) FSH, (B) LH, and (C) Testosterone Z-Scores in Male Childhood Cancer Survivors Stratified by Testicular Radiation Dose (Left) and by Cumulative Cyclophosphamide Equivalent Dose (CED, Right) **eFigure 6.** Correlations Between (A) Follicle-Stimulating Hormone (FSH), (B) Luteinizing Hormone (LH), and (C) Testosterone Z-Scores and Testicular Volume Z-Scores Measured at the Same Age **eFigure 7.** Receiver Operating Characteristic Curves and Corresponding Youden Indices (Optimal Cut-Off Points) for (A) Follicle-Stimulating Hormone (FSH) and (B) Luteinizing Hormone (LH) in Identifying Patients Non-Paternity (Left) and With Low Adult Testicular Volume (Z-Score Below -2 at Age ≥20 Years; Right) **eFigure 8.** Receiver Operating Characteristic Curves and Corresponding Youden Indices (Optimal Cut-Off Points) for Follicle-Stimulating Hormone (FSH) Measured at Age (A) 12 Years, (B) 14 Years, (C) 16 Years, and (D) 18 Years in Identifying Patients Azoospermia **eFigure 9.** Receiver Operating Characteristic Curves and Corresponding Youden Indices (Optimal Cut-Off Points) for Luteinizing Hormone (LH) Measured at Age (A) 12 Years, (B) 14 Years, (C) 16 Years, and (D) 18 Years in Identifying Patients Testosterone Replacement Therapy (TRT)

Supplement 2.
Data Sharing Statement
