# Supplementary material for: Pubertal Testicular Function in Childhood Cancer Survivors and Future Fertility
Source: JAMA Netw Open. 2026 Oct 9;9(10):e2638294. doi: 10.1001/jamanetworkopen.2026.38294 (PMC13653281; doi:10.1001/jamanetworkopen.2026.38294)
Supplement: Supplementary file 1 [file jamanetwopen-e2638294-s001.pdf]

Supplementary Online Content

Koskela M, Duffin K, Korhonen M, et al. Pubertal testicular function in childhood cancer survivors and future fertility. *JAMA Netw Open.* 2026;9(10):e2638294. doi:10.1001/jamanetworkopen.2026.38294

eTable 1. Absolute Hormone Concentrations in Male Childhood Cancer Survivors Treated at the Helsinki Children's Hospital Between 1964–2000, Stratified by Cancer Treatment Exposures
eTable 2. Multivariable Logistic Regression Analyses of Risk Factors for Testosterone Replacement Therapy in Male Childhood Cancer Survivors
eTable 3. Number of Patients at Each Timepoint in Analyses Illustrated in Figures 1–3
eFigure 1. Flow Chart of the Patient Selection Process
eFigure 2. Follicle-Stimulating Hormone (FSH) Z-Scores at Age 12–16 Years, Stratified by (A) Testicular Radiation Dose and (B) Cyclophosphamide Equivalent Dose (CED)
eFigure 3. Luteinizing Hormone (LH) Z-Scores at Age 12–16 Years, Stratified by (A) Testicular Radiation Dose and (B) Cyclophosphamide Equivalent Dose (CED)
eFigure 4. Testosterone Z-Scores at Age 12–18 Years, Stratified by (A) Testicular Radiation Dose and (B) Cyclophosphamide Equivalent Dose (CED)
eFigure 5. (A) FSH, (B) LH, and (C) Testosterone Z-Scores in Male Childhood Cancer Survivors Stratified by Testicular Radiation Dose (Left) and by Cumulative Cyclophosphamide Equivalent Dose (CED, Right)
eFigure 6. Correlations Between (A) Follicle-Stimulating Hormone (FSH), (B) Luteinizing Hormone (LH), and (C) Testosterone Z-Scores and Testicular Volume Z-Scores Measured at the Same Age
eFigure 7. Receiver Operating Characteristic Curves and Corresponding Youden Indices (Optimal Cut-Off Points) for (A) Follicle-Stimulating Hormone (FSH) and (B) Luteinizing Hormone (LH) in Identifying Patients Non-Paternity (Left) and With Low Adult Testicular Volume (Z-Score Below -2 at Age ≥20 Years; Right)
eFigure 8. Receiver Operating Characteristic Curves and Corresponding Youden Indices (Optimal Cut-Off Points) for Follicle-Stimulating Hormone (FSH) Measured at Age (A) 12 Years, (B) 14 Years, (C) 16 Years, and (D) 18 Years in Identifying Patients Azoospermia
eFigure 9. Receiver Operating Characteristic Curves and Corresponding Youden Indices (Optimal Cut-Off Points) for Luteinizing Hormone (LH) Measured at Age (A) 12 Years, (B) 14 Years, (C) 16 Years, and (D) 18 Years in Identifying Patients Testosterone Replacement Therapy (TRT)

This supplementary material has been provided by the authors to give readers additional information about their work.

© 2026 Koskela M et al. *JAMA Network Open.*

eTable 1. Absolute Hormone Concentrations in Male Childhood Cancer Survivors Treated at the Helsinki Children's Hospital Between 1964–2000, Stratified by Cancer Treatment Exposures

Age		12y	14y	16y	18y	≥20y[a]
**All patients**						
**FSH**	**Testicular radiation, Gy**					
	None	1.6 (1.1–2.1)	2.9 (2.2–6.0)	5.7 (3.2–10.4)	8.7 (4.3–17.6)	5.0 (2.6–11.6)
	>0 to <1	1.6 (0.9–2.6)	4.7 (1.3–6.1)	5.1 (3.3–9.5)	6.0 (4.2–14.2)	6.8 (3.4–12.0)
	1–14	2.7 (1.4–9.4)[b]	11.1 (5.5–17.6)[b,c]	14.6 (11.0–17.6)[b,c]	14.0 (11.4–16.2)	17.5 (12.2–24.5)[b,c]
	>14	17.8 (7.5–27.9)[b,c,d]	17.1 (16.6–30.2)	43.6 (37.0–66.8)[b,c,d]	21.8 (21.2–22.4)	34.8 (16.9–49.4)[b,c]
**LH**	**Testicular radiation, Gy**					
	None	0.8 (0.2–1.5)	2.4 (1.7–4.7)	4.6 (2.5–6.4)	5.4 (3.8–6.8)	4.3 (3.1–6.5)
	>0 to <1	1.2 (0.6–2.9)	2.7 (1.3–3.7)	3.1 (2.2–5.4)	3.8 (2.6–5.8)	4.7 (3.6–6.3)
	1–14	1.2 (0.6–2.5)	4.6 (2.9–6.3)	4.9 (4.2–7.3)	6.9 (4.8–9.3)	6.5 (5.6–10.0)[b,c]
	>14	4.8 (2.0–8.0)[b,c,d]	13.6 (7.1–20.3)	23.0 (15.6–29.7)[b,c,d]	26.8 (17.7–35.9)	15.3 (11.4–23.3)[b,c]
**T**	**Testicular radiation, Gy**					
	None	0.5 (0.3–2.2)	5.4 (3.1–11.2)	12.5 (9.8–17.6)	16.0 (13.1–22.0)	14.0 (11.6–18.9)
	>0 to <1	1.4 (0.6–5.3)	6.3 (2.7–10.2)	12.0 (8.8–16.0)	12.0 (11.0–18.0	12.9 (10.0–18.2)
	1–14	1.5 (0.4–3.5)	8.3 (5.5–11.4)	11.5 (8.2–13.7)	15.0 (11.3–17.4)	15.2 (12.1–18.4)
	>14	0.3 (0.3–1.2)	4.6 (0.6–12.5)	6.3 (4.3–12.0)	14.7 (13.4–16.0)	13.0 (3.6–19.4)
**Patients with testicular radiation ≥1 Gy excluded**						
**FSH**	**CED, g/m²**					
	<4	1.7 (1.1–2.1)	2.4 (1.8–3.2)	3.8 (2.8–5.4)	4.6 (2.2–8.3)	4.0 (2.2–6.6)
	≥4 to <12	1.9 (1.1–3.2)	5.2 (3.6–6.8)[e]	5.7 (3.3–10.4)	9.6 (5.7–19.2)[e]	6.4 (3.1–11.2)
	≥12	1.3 (0.8–1.8)	6.0 (2.4–8.3)	9.9 (6.4–16.4)[e]	10.9 (6.2–18.7)[e]	11.1 (7.0–18.4)[e,f]
**LH**	**CED, g/m²**					
	<4	0.6 (0.4–1.0)	1.8 (1.2–2.3)	3.6 (2.5–5.0)	4.0 (3.3–5.3)	3.5 (2.9–5.4)
	≥4 to <12	3.5 (1.4–5.0)[e]	3.2 (2.7–5.1)[e]	4.8 (2.3–6.2)	5.0 (3.5–6.8)	4.6 (3.7–5.8)
	≥12	0.9 (0.3–1.5)	2.9 (1.6–4.3)	5.3 (2.4–7.3)	6.0 (3.4–6.9)	5.3 (4.3–7.3)[e]
**T**	**CED, g/m²**					
	<4	0.5 (0.3–1.1)	5.4 (4.2–11.0)	16.0 (10.4–17.7)	16.0 (11.5–21.4)	13.0 (11.0–19.0)
	≥4 to <12	2.9 (0.6–5.3)	7.1 (2.3–10.3)	10.2 (8.8–19.5)	15.0 (13.0–19.0)	16.9 (11.7–18.8)
	≥12	1.0 (0.3–2.7)	5.2 (2.0–13.7)	12.0 (8.8–13.4)	14.0 (12.0–19.0)	13.0 (10.0–18.3)

CED, cyclophosphamide equivalent dose; Gy, gray; T, testosterone; y, years
Data reported as median (interquartile range)
FSH and LH reported as IU/L and testosterone as nmol/L
[a]Median age at measurement 25 (interquartile range 20–28) years
[b]Significant difference (p<0.05) when compared with hormone concentration in Testicular radiation group "None" in the same time point
[c]Significant difference (p<0.05) when compared with hormone concentration in Testicular radiation group ">0 to <1" in the same time point
[d]Significant difference (p<0.05) when compared with hormone concentration in Testicular radiation group "1–14" in the same time point
[e]Significant difference (p<0.05) when compared with hormone concentration in CED group "<4" in the same time point
[f]Significant difference (p<0.05) when compared with hormone concentration in CED group "≥4 to <12" in the same time point

© 2026 Koskela M et al. *JAMA Network Open.*

eTable 2. Multivariable Logistic Regression Analyses of Risk Factors for Testosterone Replacement Therapy in Male Childhood Cancer Survivors
Analyses were conducted in the full survivor cohort (N=255) treated at the Helsinki Children's Hospital between 1964–2000.

	**TRT**[a] OR (95% CI)
Cumulative testicular radiation dose[b], Gy	
0	1
>0 to <1	0.62 (0.25–1.45)
≥1 to ≤14	**3.13 (1.44–6.92)**
>14	**126.28 (22.68–2401.08)**
CED, g/m²	
<4	1
≥4 to <12	1.76 (0.78–3.98)
≥12	1.77 (0.82–3.90)
Cumulative pituitary radiation dose[c], Gy	
<20	1
≥20	1.03 (0.43–2.33)

TRT, testosterone replacement therapy; CED, cyclophosphamide equivalent dose; OR, odds ratio
Bold OR values denote p<0.05.
[a]Initiated during puberty or in adulthood
[b]Median (interquartile range) testicular radiation dose in <1 Gy group was 0.1 (0.04–0.2) Gy, in ≥1 to ≤14 Gy group 10 (10–11) Gy, and in >14 Gy group 24 (24–24) Gy.
[c]Median (interquartile range) pituitary radiation dose in <20 Gy group was 0.1 (0–10) Gy and in ≥20 Gy group 24 (24–24) Gy.

© 2026 Koskela M et al. *JAMA Network Open.*

eTable 3. Number of Patients at Each Timepoint in Analyses Illustrated in Figures 1–3

Age		12y	14y	16y	18y	≥20y[a]		12y	14y	16y	18y	≥20y[a]
**Figure 1A - left**							**Figure 1A - right**					
	**Testicular radiation, Gy**						**CED, g/m^2**					
**FSH**	None	33	31	45	30	59	<4	26	20	22	21	38
	>0 to <1	16	15	16	24	30	≥4 to <12	10	12	19	12	22
	1–14	23	16	19	15	15	≥12	13	14	20	21	29
	>14	11	3	5	2	8						
**Figure 1B - left**							**Figure 1B - right**					
	**Testicular radiation, Gy**						**CED, g/m^2**					
**LH**	None	30	32	47	30	59	<4	24	21	24	20	38
	>0 to <1	16	15	16	24	30	≥4 to <12	9	12	19	13	22
	1–14	22	16	19	14	15	≥12	13	14	20	21	29
	>14	10	4	5	2	8						
**Figure 1C - left**							**Figure 1C - right**					
	**Testicular radiation, Gy**						**CED, g/m^2**					
**T**	None	32	31	42	29	57	<4	23	19	21	19	37
	>0 to <1	13	14	17	25	31	≥4 to <12	10	13	16	13	23
	1–14	21	16	17	14	15	≥12	12	13	22	22	28
	>14	9	4	5	2	6						
**Figure 2A - left**							**Figure 2A - right**					
	**Semen analysis**						**TRT**					
	Normosp.	18	11	18	13	35						
**FSH**	Oligosp.	3	3	7	6	14	No	62	59	72	64	97
	Azoosp.	4	3	8	11	25	Yes	20	7	13	8	16
**Figure 2B - left**							**Figure 2B - right**					
	**Semen analysis**						**TRT**					
	Normosp.	16	12	19	14	34						
**LH**	Oligosp.	3	3	7	6	15	No	58	60	74	63	97
	Azoosp.	3	3	8	11	24	Yes	19	8	13	8	16
**Figure 2C - left**							**Figure 2C - right**					
	**Semen analysis**						**TRT**					
	Normosp.	20	13	17	14	35						
**T**	Oligosp.	3	3	7	4	15	No	57	57	68	62	95
	Azoosp.	4	3	8	11	25	Yes	18	8	13	8	14
**Figure 3A - left**							**Figure 3A - right**					
	**Paternity**						**TV[b]**					
**FSH**	No	54	45	56	39	51	< –2	7	2	8	4	10
	Yes	28	21	29	33	62	> –2	8	10	15	10	29
**Figure 3B - left**							**Figure 3B - right**					
	**Paternity**						**TV[b]**					
**LH**	No	51	46	57	38	52	< –2	7	2	7	4	10
	Yes	26	22	30	33	61	> –2	7	11	16	11	30
**Figure 3C - left**							**Figure 3C - right**					
	**Paternity**						**TV[b]**					
**T**	No	50	44	53	38	47	< –2	7	3	7	3	10
	Yes	25	21	28	32	62	> –2	10	11	15	10	30

CED, cyclophosphamide equivalent dose; Gy, gray; TV, testicular volume (measured at age ≥20); T, testosterone; y, years; TRT, testosterone replacement therapy

[a]Median age at measurement 25 (interquartile range 20–28) years

[b]Groups denote testicular volumes over or under –2 SD scores. See Methods for details.

© 2026 Koskela M et al. *JAMA Network Open.*

eFigure 1. Flow Chart of the Patient Selection Process
Exclusion criteria at each timepoint were non-availability (NA) of hormone samples in hospital records or ongoing testosterone replacement therapy (TRT).

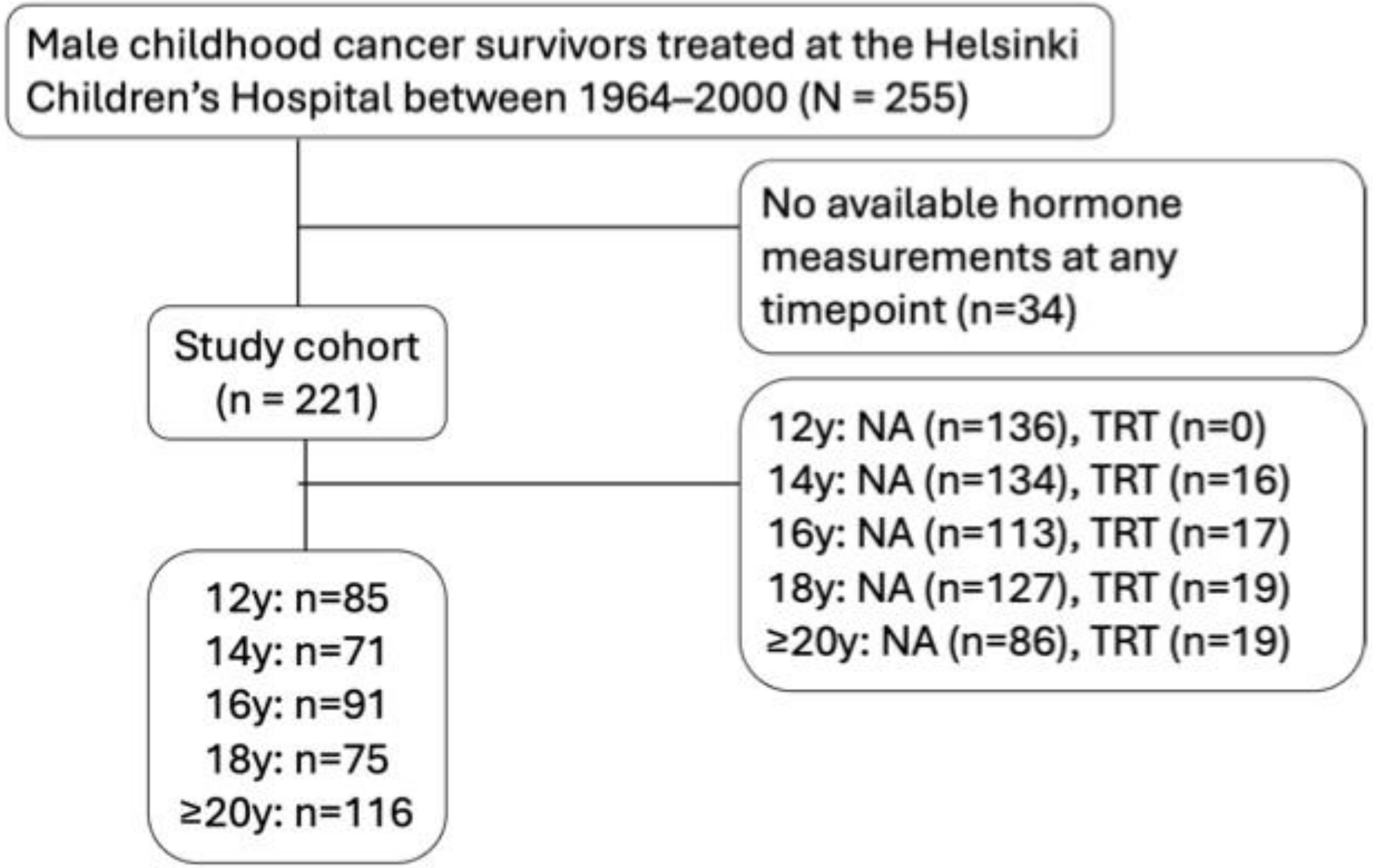

© 2026 Koskela M et al. *JAMA Network Open.*

eFigure 2. Follicle-Stimulating Hormone (FSH) Z-Scores at Age 12–16 Years, Stratified by (A) Testicular Radiation Dose and (B) Cyclophosphamide Equivalent Dose (CED)

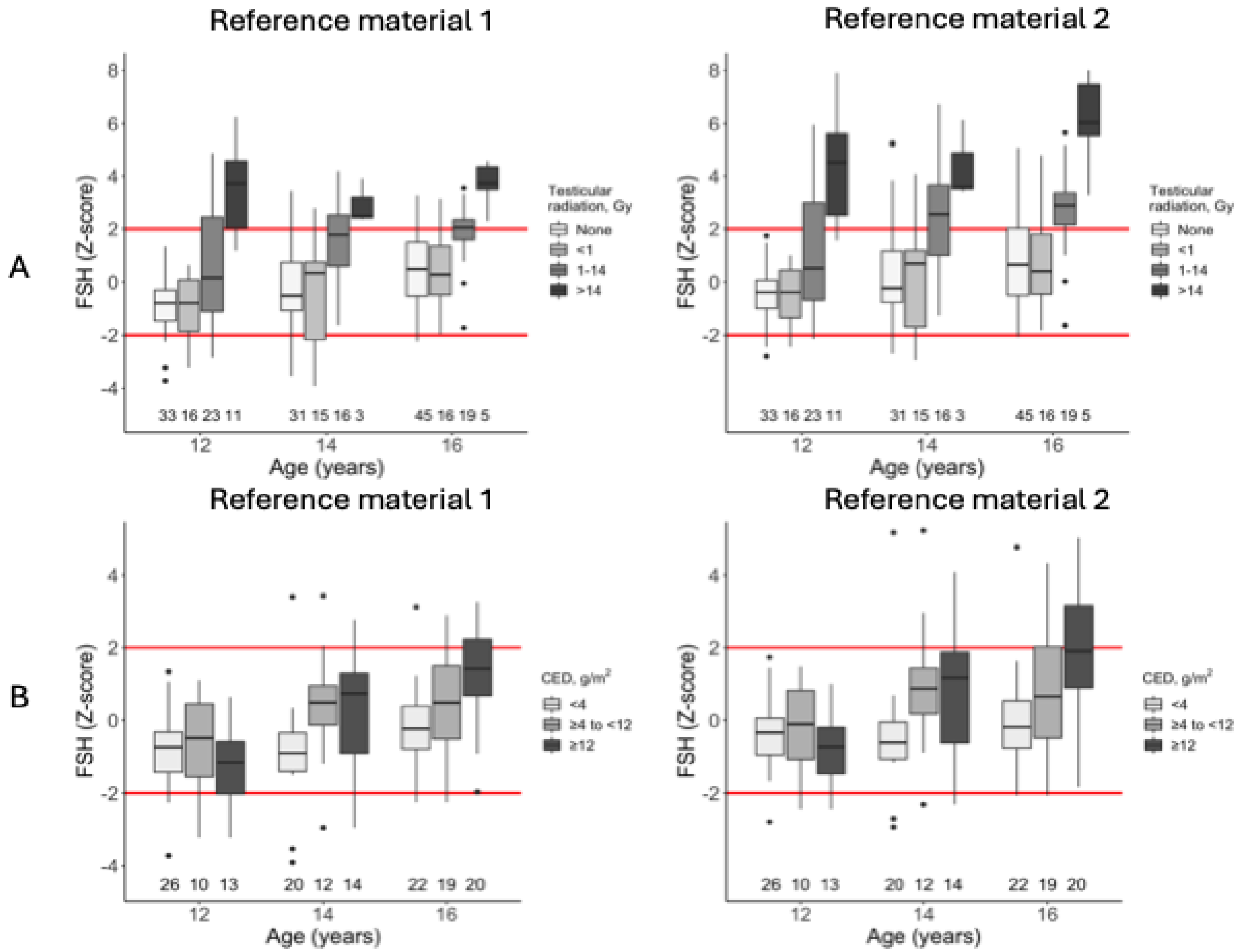

Values were standardized using Reference material 1 (left) and Reference material 2 (right). Patients with testicular irradiation ≥1 Gy were excluded from the analyses involving CED. Area between the two horizontal red lines indicates normal reference range. See Methods for details on the Reference materials.

© 2026 Koskela M et al. *JAMA Network Open.*

eFigure 3. Luteinizing Hormone (LH) Z-Scores at Age 12–16 Years, Stratified by (A) Testicular Radiation Dose and (B) Cyclophosphamide Equivalent Dose (CED)

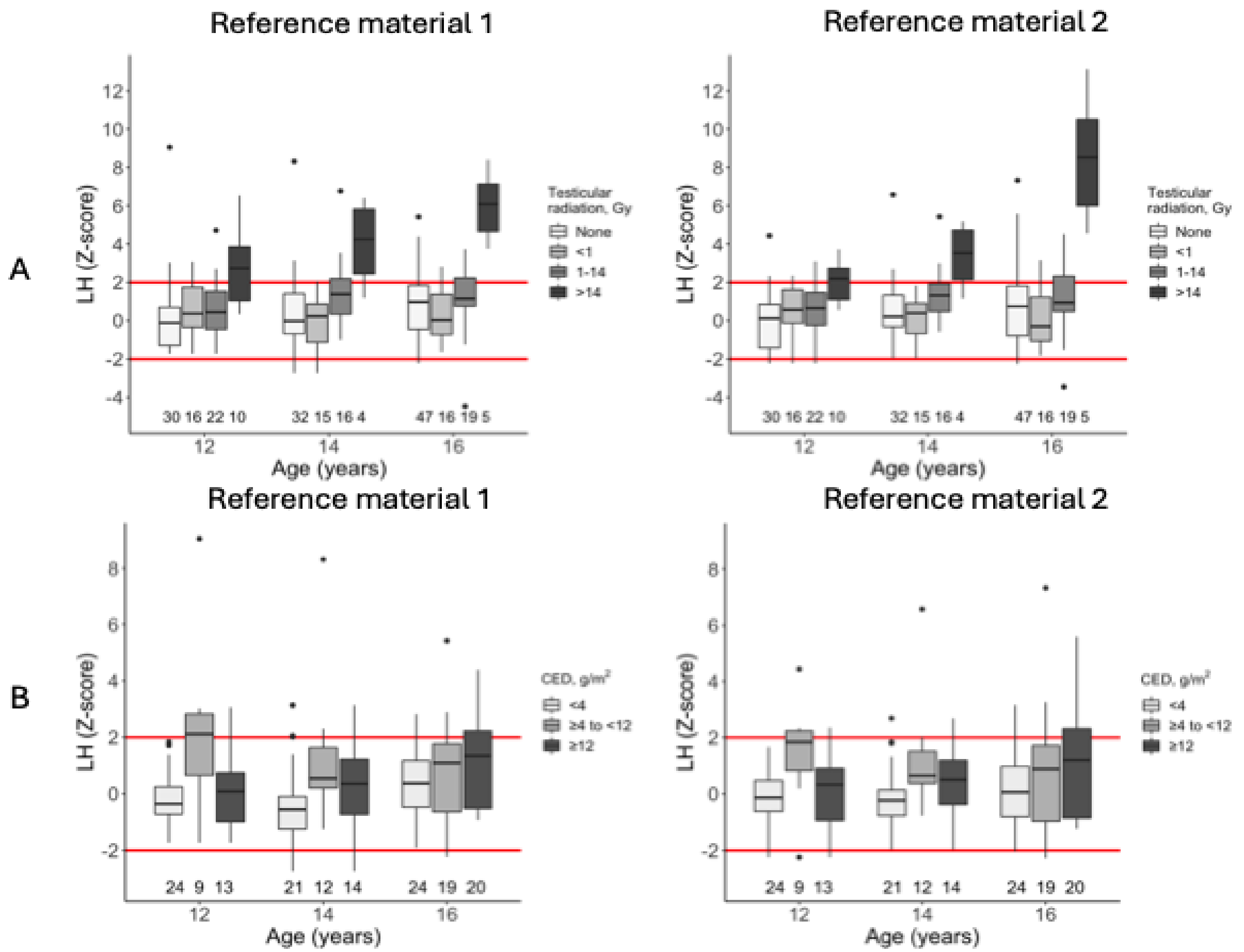

Values were standardized using Reference material 1 (left) and Reference material 2 (right). Patients with testicular irradiation ≥1 Gy were excluded from the analyses involving CED. Area between the two horizontal red lines indicates normal reference range. See Methods for details on the Reference materials.

© 2026 Koskela M et al. *JAMA Network Open.*

eFigure 4. Testosterone Z-Scores at Age 12–18 Years, Stratified by (A) Testicular Radiation Dose and (B) Cyclophosphamide Equivalent Dose (CED)

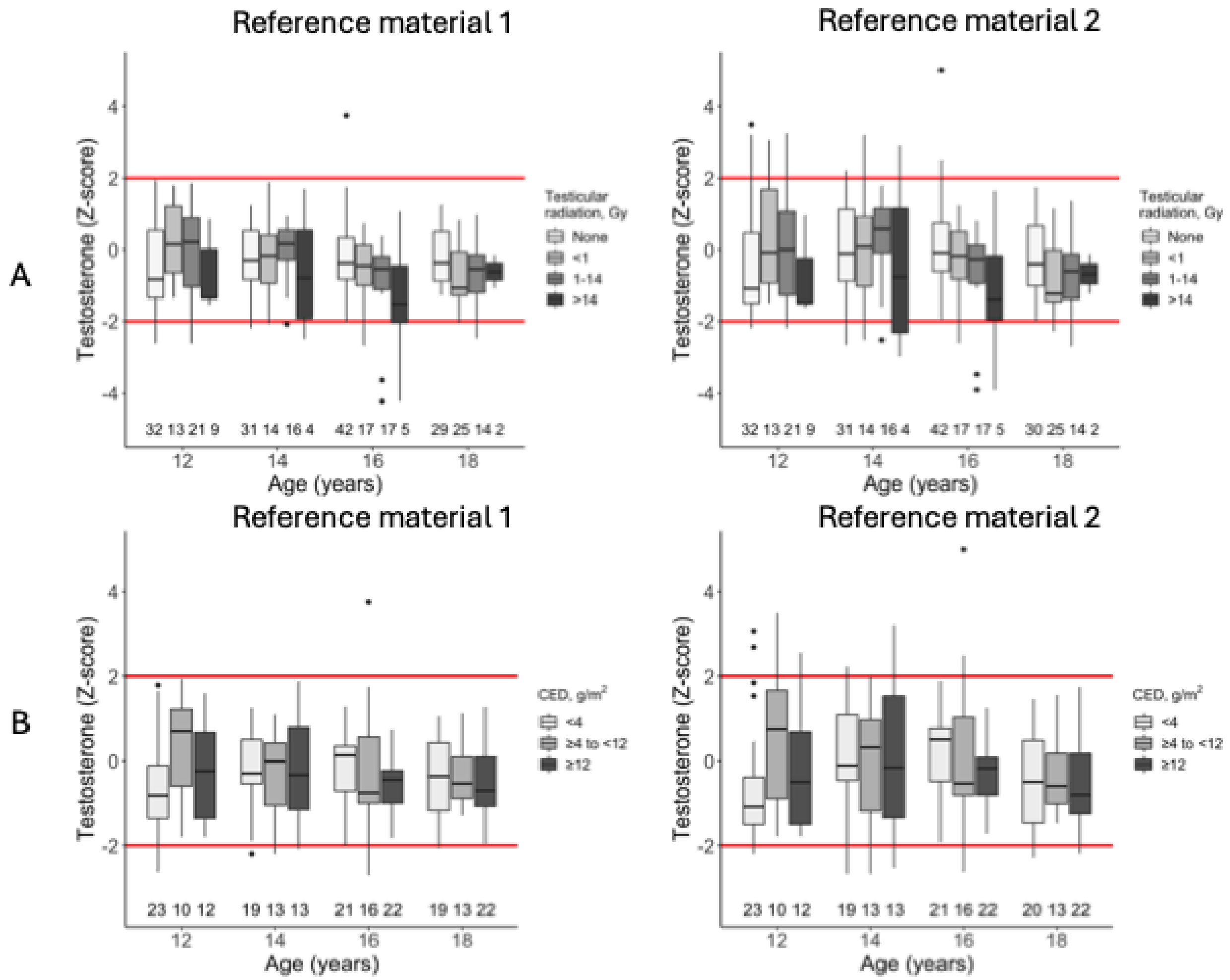

Values were standardized using Reference material 1 (left) and Reference material 2 (right). Patients with testicular irradiation ≥1 Gy were excluded from the analyses involving CED. Area between the two horizontal red lines indicates normal reference range. See Methods for details on the Reference materials.

© 2026 Koskela M et al. *JAMA Network Open.*

eFigure 5. (A) FSH, (B) LH, and (C) Testosterone Z-Scores in Male Childhood Cancer Survivors Stratified by Testicular Radiation Dose (Left) and by Cumulative Cyclophosphamide Equivalent Dose (CED, Right)

Patients with pituitary irradiation ≥ 20 Gy were excluded from all analyses, and those with testicular irradiation ≥ 1 Gy were excluded from the analyses involving CED. Area between the two horizontal red lines indicates normal reference range. Hormone values were standardized to age-specific Z-scores using Reference material 1. The median age at measurement in the ≥20-year group was 25 (interquartile range 20–28) years.

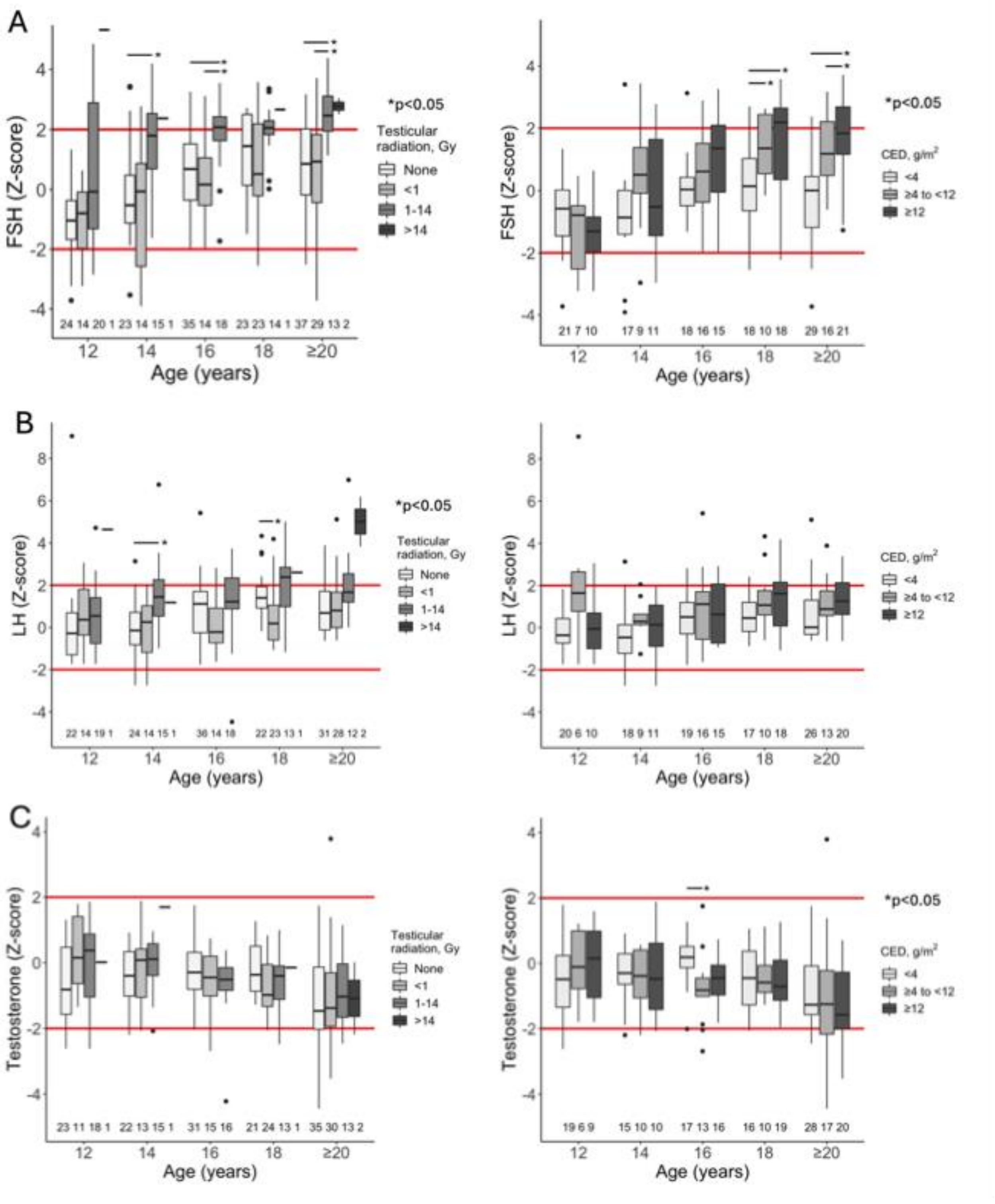

© 2026 Koskela M et al. *JAMA Network Open.*

eFigure 6. Correlations Between (A) Follicle-Stimulating Hormone (FSH), (B) Luteinizing Hormone (LH), and (C) Testosterone Z-Scores and Testicular Volume Z-Scores Measured at the Same Age
Hormone values were standardized to age-specific Z-scores using Reference material 1.

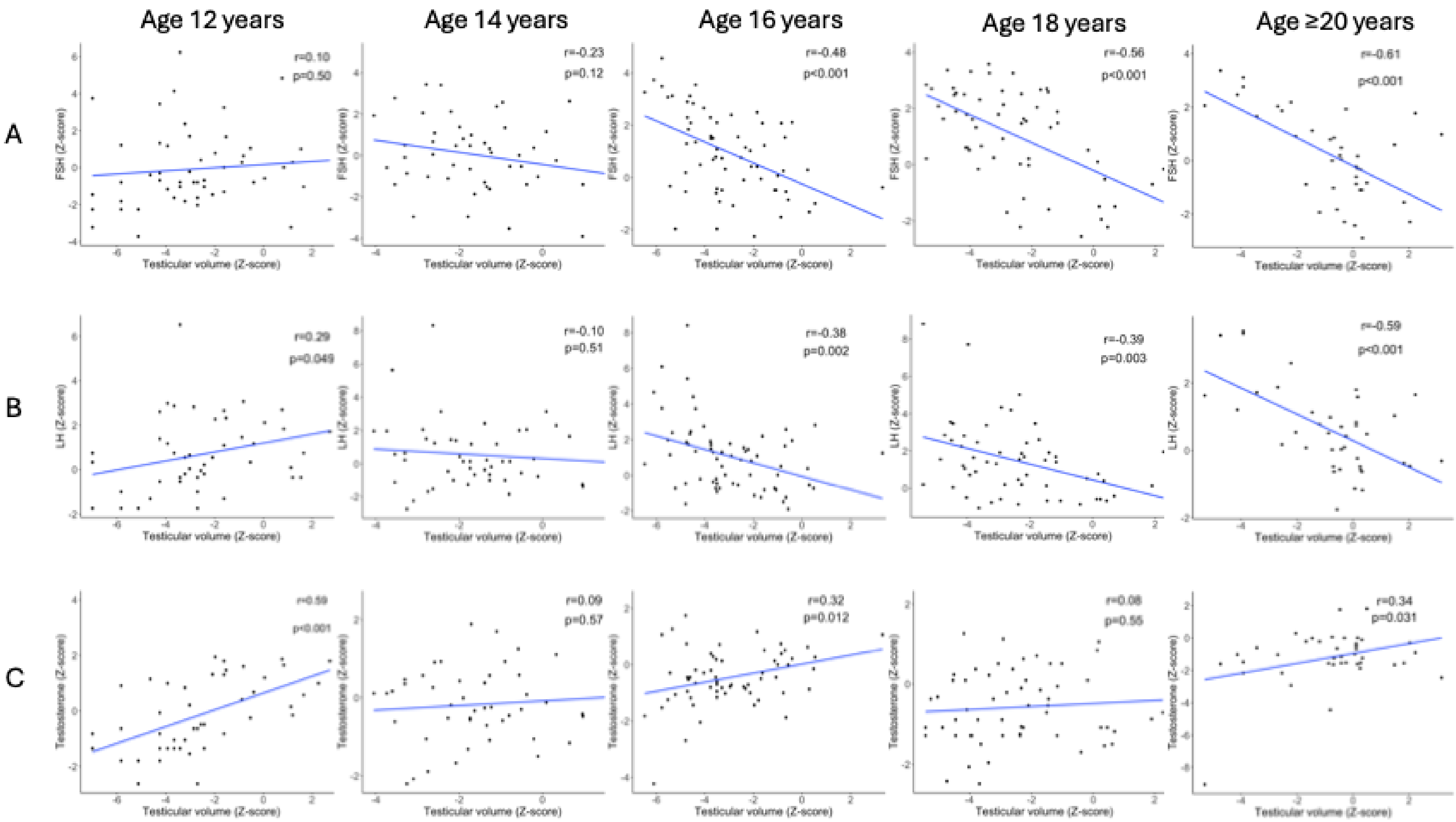

© 2026 Koskela M et al. *JAMA Network Open.*

eFigure 7. Receiver Operating Characteristic Curves and Corresponding Youden Indices (Optimal Cut-Off Points) for (A) Follicle-Stimulating Hormone (FSH) and (B) Luteinizing Hormone (LH) in Identifying Patients Non-Paternity (Left) and With Low Adult Testicular Volume (Z-Score Below -2 at Age ≥20 Years; Right)
Hormone values were standardized to age-specific Z-scores using Reference material 1.

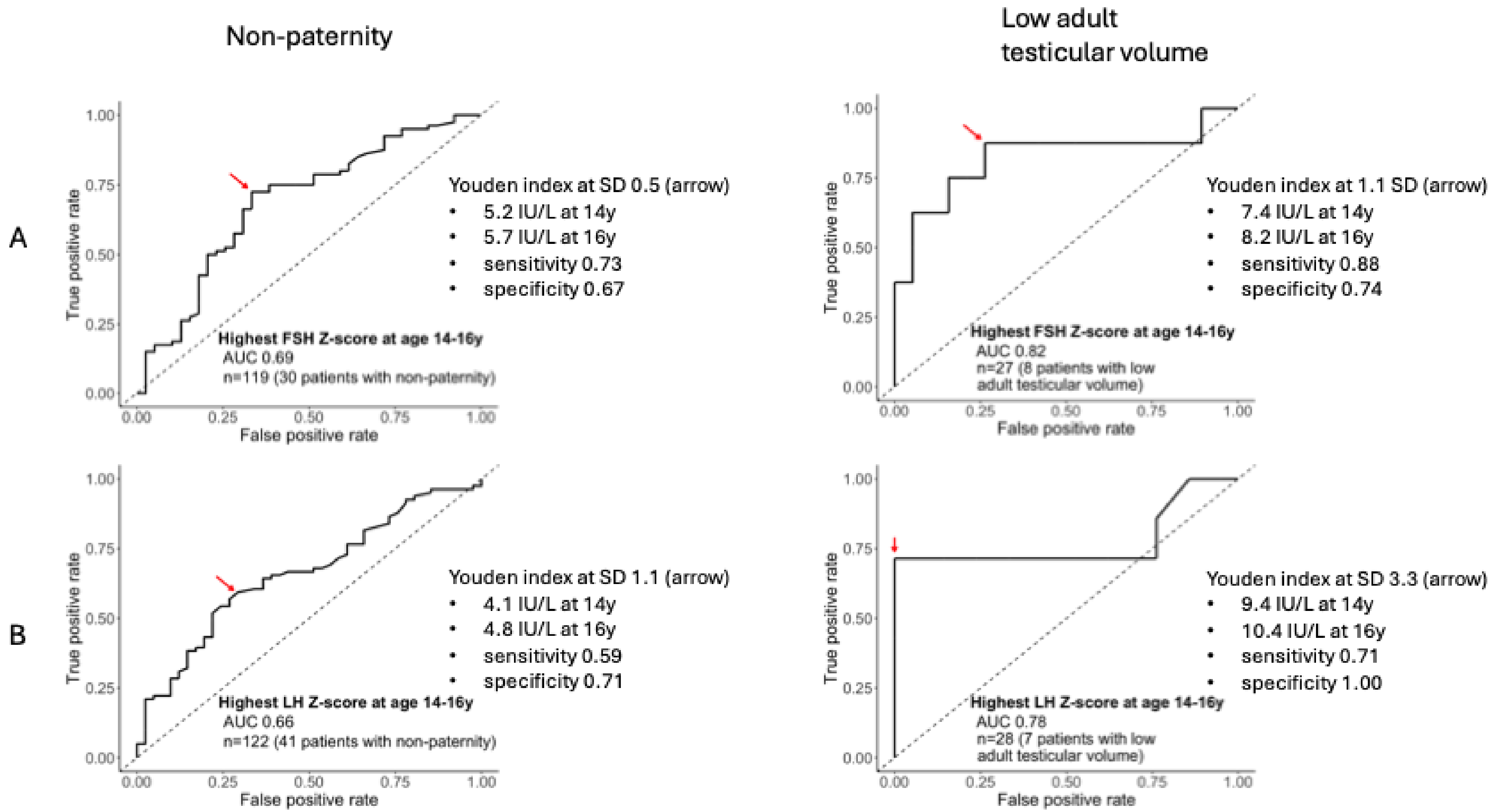

© 2026 Koskela M et al. *JAMA Network Open.*

eFigure 8. Receiver Operating Characteristic Curves and Corresponding Youden Indices (Optimal Cut-Off Points) for Follicle-Stimulating Hormone (FSH) Measured at Age (A) 12 Years, (B) 14 Years, (C) 16 Years, and (D) 18 Years in Identifying Patients Azoospermia
Hormone values were standardized to age-specific Z-scores using Reference material 1.

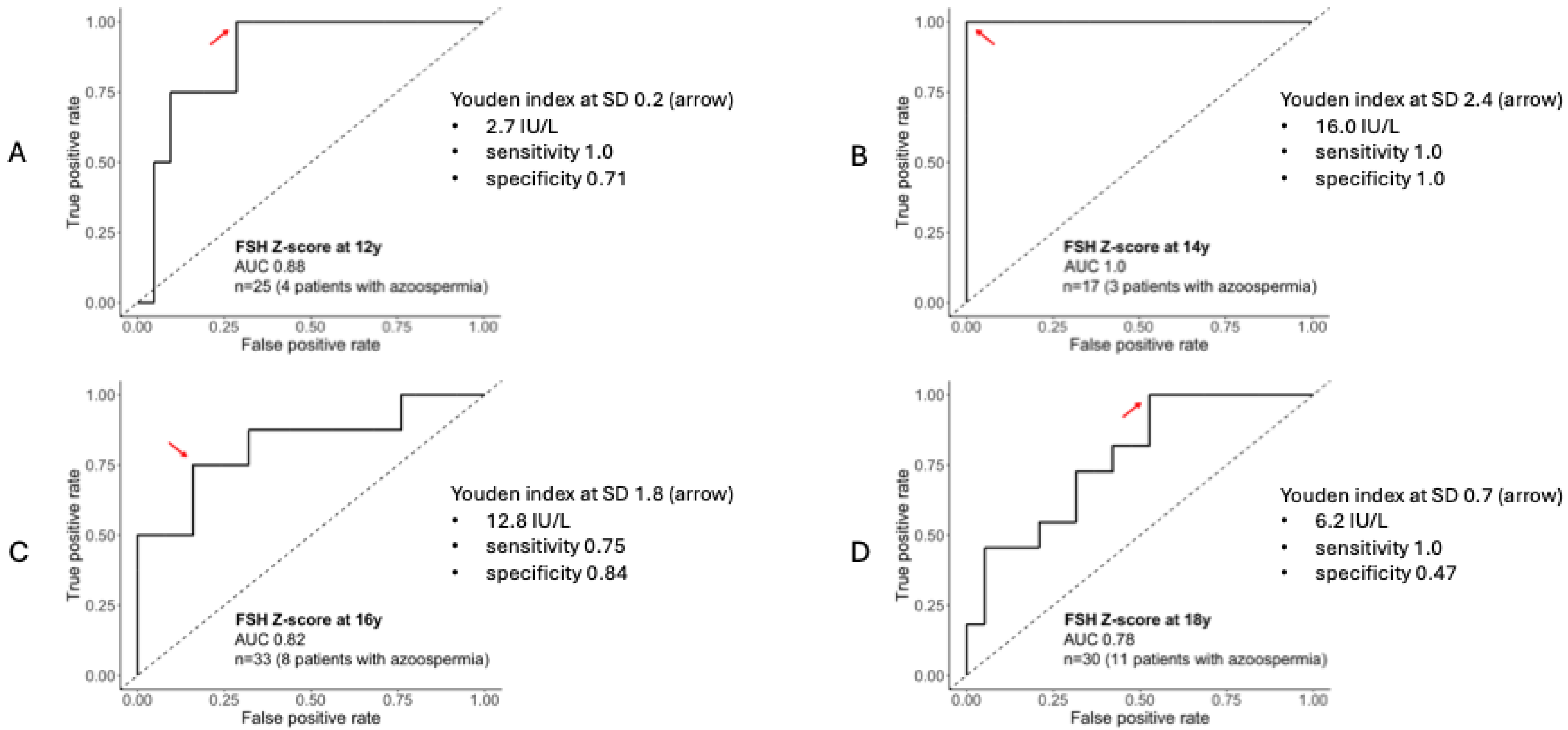

© 2026 Koskela M et al. *JAMA Network Open.*

eFigure 9. Receiver Operating Characteristic Curves and Corresponding Youden Indices (Optimal Cut-Off Points) for Luteinizing Hormone (LH) Measured at Age (A) 12 Years, (B) 14 Years, (C) 16 Years, and (D) 18 Years in Identifying Patients Testosterone Replacement Therapy (TRT)
Hormone values were standardized to age-specific Z-scores using Reference material 1.

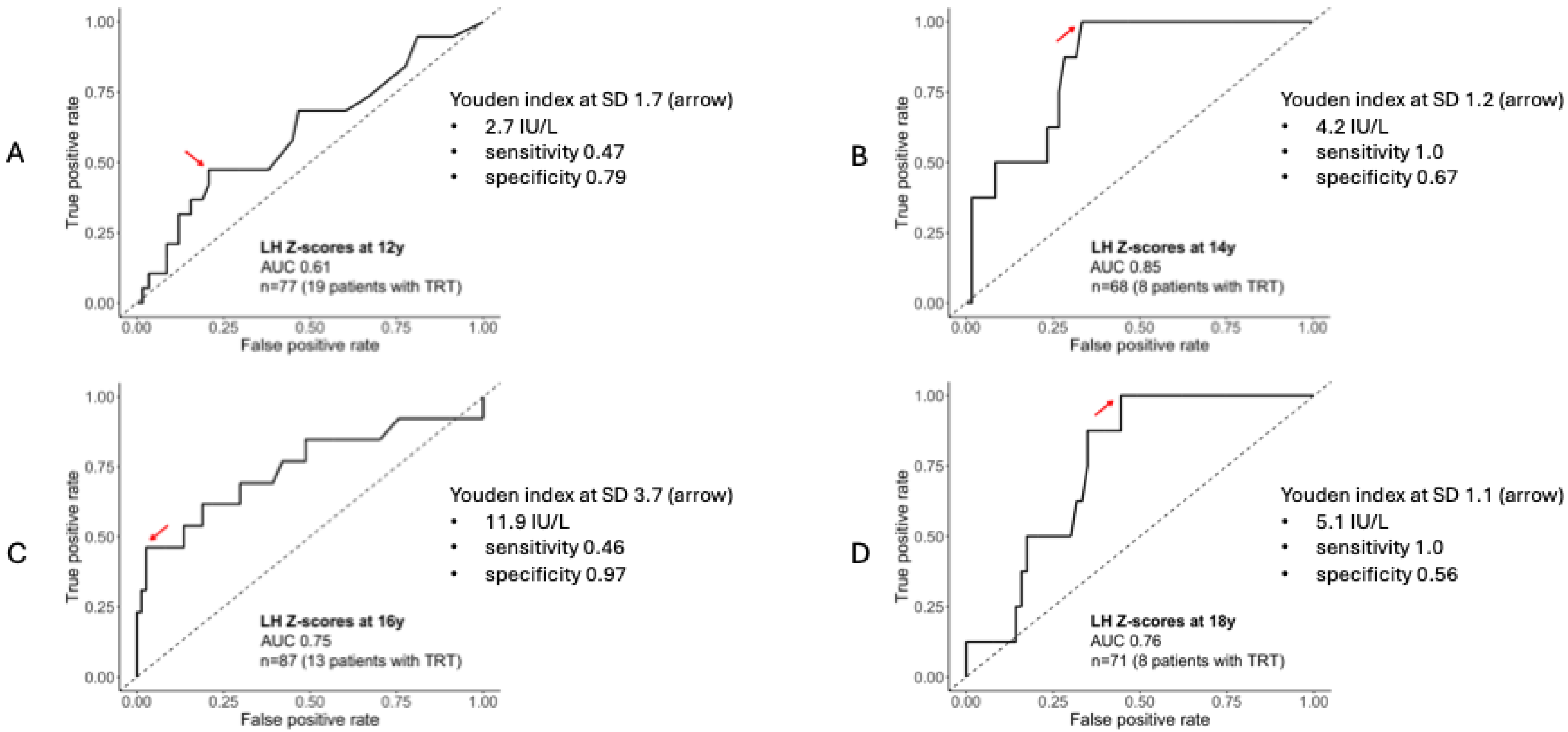

© 2026 Koskela M et al. *JAMA Network Open.*
