# Supplementary material for: Pubertal Testicular Function in Childhood Cancer Survivors and Future Fertility
Source: JAMA Netw Open. 2026 Oct 9;9(10):e2638294. doi: 10.1001/jamanetworkopen.2026.38294 (PMC13653281; doi:10.1001/jamanetworkopen.2026.38294)
Supplement: Supplementary file 2 [file jamanetwopen-e2638294-s002.pdf]

Data Sharing Statement

Koskela. Pubertal Testicular Function in Childhood Cancer Survivors and Future Fertility.
JAMA Netw Open. Published October 09, 2026. doi:10.1001/jamanetworkopen.2026.38294

Data
Data available: Additional data available on reasonable request
